# Effects of doping and annealing on properties of ZnO films grown by atomic layer deposition

**DOI:** 10.1186/s11671-015-0801-y

**Published:** 2015-02-18

**Authors:** Aiji Wang, Tingfang Chen, Shuhua Lu, Zhenglong Wu, Yongliang Li, He Chen, Yinshu Wang

**Affiliations:** Department of Physics, Beijing Normal University, Beijing, 100875 China; Analytical and Testing Center, Beijing Normal University, Beijing, 100875 China; School of Police Information Engineering, People’s Public Security University of China, Beijing, 100038 China

**Keywords:** ZnO films, Al doping, ALD, Optical properties, Electrical properties, Annealing atmospheres

## Abstract

Undoped and Al-doped ZnO films were synthesized by atomic layer deposition at 150°C and then annealed at 350°C in different atmospheres. Effects of doping and annealing on the film growth mode and properties were investigated. The undoped film has strong UV emission and weak Zn interstitial emission. Annealing introduces O vacancies, decreases Zn interstitials, and results in weakening and blue-shifting of the UV emission which is sensitive to annealing atmosphere. Al doping induces the film growing with its c-axis parallel to the substrate surface. It also introduces non-radiative centers and weakens the UV emission. Al doping widens the film bandgap, which has a quadratic dependence on Al content. Al doping decreases the film resistivity to 5.3 × 10^−3^ Ω · cm. Annealing has little effect on photoluminescence of the doped films, but it degrades undoped and doped ZnO film conductivity dramatically; and the degradation depends on the annealing ambient.

## Background

Transparent conducting oxide (TCO) plays a significant role in transparent devices, such as solar cell panels, flat panel displays, and organic light-emitting diodes [1]. So far, indium tin oxide (ITO) is a typical commercial TCO. It yields a low resistivity of 10^−4^ Ω · cm, has a transmittance higher than 85%, and possesses good etch-ability [2]. However, the scarce and toxic nature of indium and instability of ITO have stimulated researchers to explore alternative TCO materials for ITO [3,4]. ZnO is a wide bandgap semiconductor, which has potential applications in the fields of ultraviolet light emitters, photosensitizers, optoelectronics, gas sensors, etc. [5]. In recent years, ZnO films doped with group-III elements have attracted considerable attention as a candidate for TCO [6-13]. Among them, Al-doped ZnO is one of the most prospective alternative candidates for TCO since Al is abundant and nontoxic [4]. Various methods such as spray pyrolysis [9], atomic layer deposition (ALD) [10], magnetron sputtering [11], chemical vapor deposition [12], and pulsed laser deposition [13] have been adopted to deposit Al-doped ZnO films. The qualities of the films are sensitive to growth techniques and parameters. Compared with other techniques, ALD could deposit uniform and conformal film on large areas at low growth temperature. In addition, the thickness of the film could be controlled accurately. Effects of ALD process parameters such as growth temperature, purge length, and the precursor expose time on the properties of Al-doped ZnO films have been reported [10,14-18]. However, there are seldom reports on thermal stability and property evolution of Al-doped ZnO films grown by ALD after post annealing. Post annealing and the annealing atmospheres are crucial for the film properties [19-21]. Kim et al. [19] observed an increase of carrier concentration in Al-doped ZnO films grown by magnetron sputtering after annealing in vacuum. Lin et al. [20] observed a decrease in carrier concentration of heavily Al-doped ZnO films grown by similar method after annealing in N_2_ and O_2_ atmosphere. Zhou et al. [21] observed an improvement of conductivity of Al-doped ZnO films grown by magnetron sputtering after annealing in a mixture of N_2_ and O_2_. The results of different groups are controversial, and the related mechanisms are still unclear. Furthermore, ZnO films grown by different methods would show different property evolution when they are annealed under the same conditions. The stability of Al-doped ZnO films is also important for the technology of electronic and optoelectronic devices. It needs to be investigated further.

In this work, undoped and Al-doped ZnO films were deposited on glass substrates by ALD. The films were annealed at 350°C in Ar, N_2_, and air atmosphere, separately. Effects of doping and post annealing on the film growth mode, bandgap evolution, and optical and electrical properties were investigated in details.

## Methods

Undoped and Al-doped ZnO films were deposited on glass slides in a SUMALE™ ALD R200 reactor. Precursors for Zn, Al, Mg, and oxygen were diethylzinc (DEZ), trimethylaluminum (TMA), magnesocene (MS), and H_2_O, respectively. High purity nitrogen (N_2_) was used as both the carrier and purge gas. DEZ-H_2_O cycles are chosen for depositing ZnO films and TMA-H_2_O and MS-H_2_O cycles are for Al and Mg doping. All pulse times for DEZ, TMA, MS, and H_2_O were kept at 0.1 s, and the purging time was kept at 6 s. The growth temperature was kept at 150°C. To achieve the desired compositions, a single TMA-H_2_O cycle or MS-H_2_O cycle was inserted after a set number (*n*) of DEZ-H_2_O cycles. *n* was chosen to be 48, 24, 16, and 12 for Al-doped films. Al concentration in the film was denoted as the ideal concentration, which was calculated according to that reported in the reference [10]. The thickness of all films was controlled at about 100 nm by choosing the number of total cycles. Annealing process of the films was performed in a quartz tube furnace at 350°C in Ar, N_2_, and air atmosphere, respectively.

The structures of the films were analyzed by an X-ray diffractometer (SHIMADZU XRD-6000) with a Cu-Kα radiation. Surface morphologies of the films were observed by a scanning electron microscope (SEM, HITACHI S-4800). The absorption spectra were measured by an UV-1900 spectrometer. Photoluminescence (PL) spectra of the films were recorded by a Jobin-Yvon micro-Raman spectrometer using a 325-nm He-Cd laser as an excitation source. The electrical properties of the films were measured on a SCS-4200 system, utilizing a four-point Van der Pauw contact configuration. All measurements were performed at room temperature.

## Results and discussion

### Structure and morphology evolution

**Figure 1:**
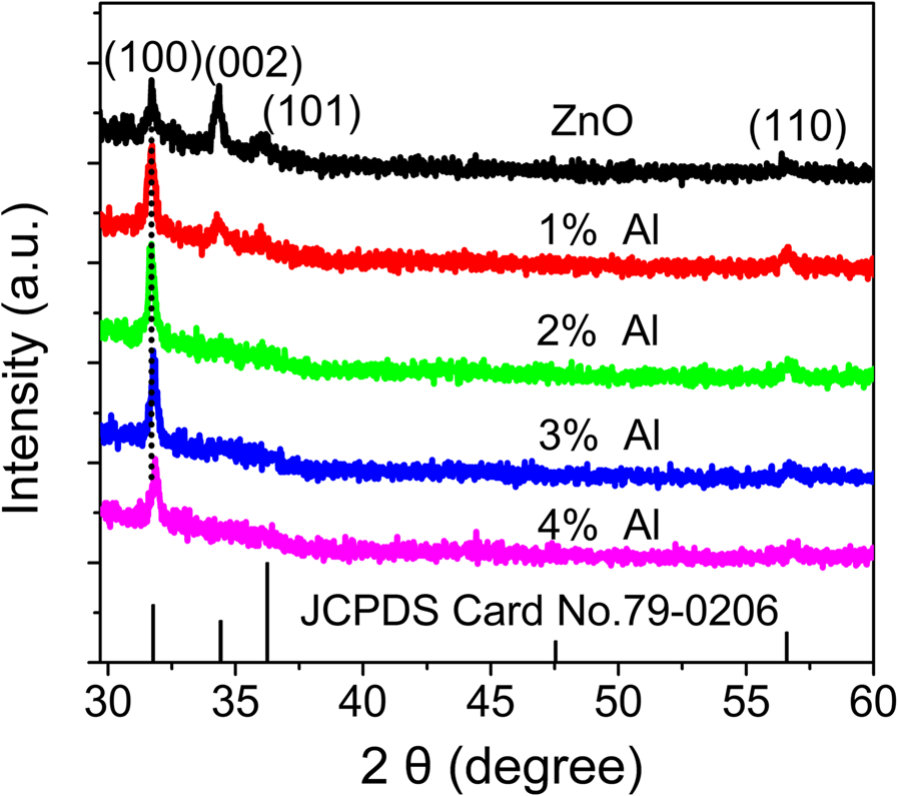
**XRD patterns of undoped and Al-doped ZnO films.**

**Figure 2:**
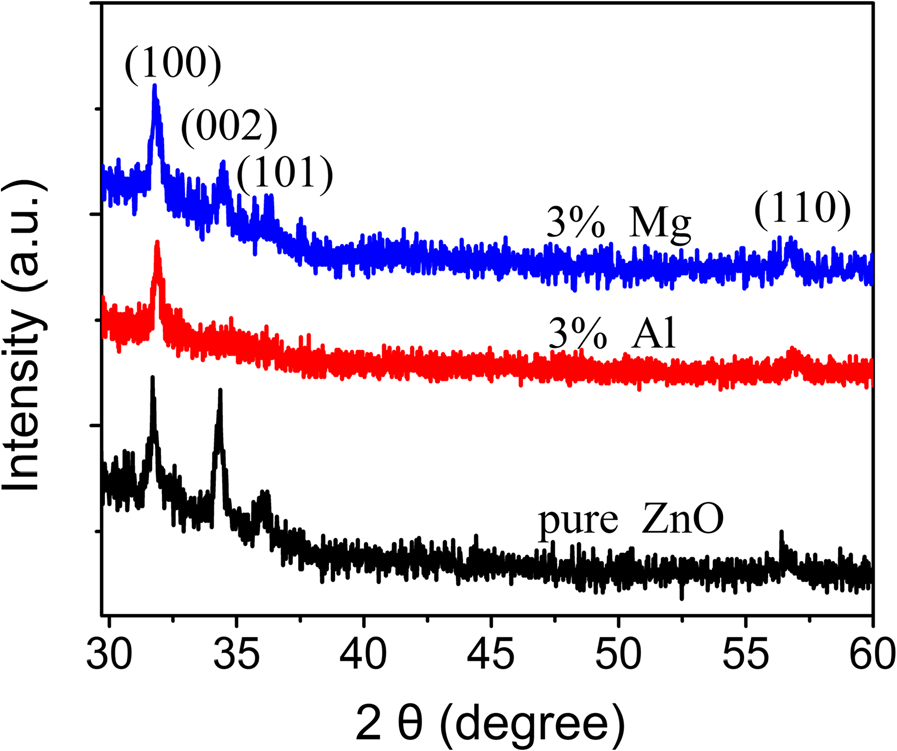
**XRD patterns of undoped, 3 at.% Al-doped, and 3 at.% Mg-doped ZnO films.**

XRD patterns of the as-grown undoped and Al-doped ZnO films are shown in Figure 1. Standard spectrum of ZnO is also shown in Figure 1 (JCPDS card no. 79-0206). Diffraction peaks are observed at 31.8°, 34.5°, 36.0°, and 56.5° in the undoped film, which can be indexed as diffractions of (100), (002), (101), and (110) planes of wurtzite-structured ZnO with lattice constant of *a* = 0.325, *c* = 0.521 nm. Compared with the standard spectrum of ZnO, the diffraction intensity of (002) planes is much stronger. This suggests that crystal c-axis of the undoped ZnO film is inclined to be perpendicular to the substrate surface. Once the film is doped with Al, the diffraction from (100) planes enhances dramatically. With an increase in Al content, the diffraction from (002) planes becomes unobserved and only the strong diffraction peak of (100) planes and weak diffraction peak of (110) planes are observed. This suggests that Al doping affects the growth mode of ZnO films. Similar Al doping effects on the growth mode have been reported in references [10,14,15]. Banerjee et al. [10] attributed the enhanced diffraction of (100) planes to the preferential growth of (100) planes, which was due to the disturbance of the charge neutrality of (100) planes induced by substitution of Zn^2+^ by Al^3+^ ions. To investigate whether it is the disturbance of the charge neutrality that affects the growth mode, 3 at.% Mg-doped ZnO film was also grown at the same temperature using MS and H_2_O as doping precursors. The XRD spectra of undoped and Mg- and Al-doped films are shown in Figure 2. Similar to that observed in Al-doped films, the dominant diffraction of Mg-doped film is also from (100) planes. Substitution of Zn^2+^ by Mg^2+^ would not affect the charge neutrality of the (100) planes. The surface-free energy of (002) planes of wurtzite-structured ZnO is the lowest [22]. Therefore, ZnO usually grows preferentially along the c-axis. The decomposing temperature of TMA and MS is much higher than that of DEZ. During the growing of undoped films at 150°C, DEZ could decompose easily and the redundant clusters would be removed efficiently. ZnO nuclei could adsorb the precursor molecules for the further growth, and the grains would grow preferentially with c-axis inclining to be perpendicular to the surface. Once TMA and MS are introduced for Al or Mg doping, the adsorbed TMA and MS molecules could not release their redundant clusters efficiently. The further growth would be disturbed, and the growth rate would be lower [23]. Then, the grains would grow with c-axis parallel to the substrate surface and diffraction from (100) planes would be enhanced. Otherwise, it can also be seen from Figures 1 and 2 that the diffraction peak of (100) planes of doped films shifts to higher angle. This means that Zn^2+^ ions are replaced by Al^3+^ ions, resulting in the shrinkage of the lattice.

**Figure 3:**
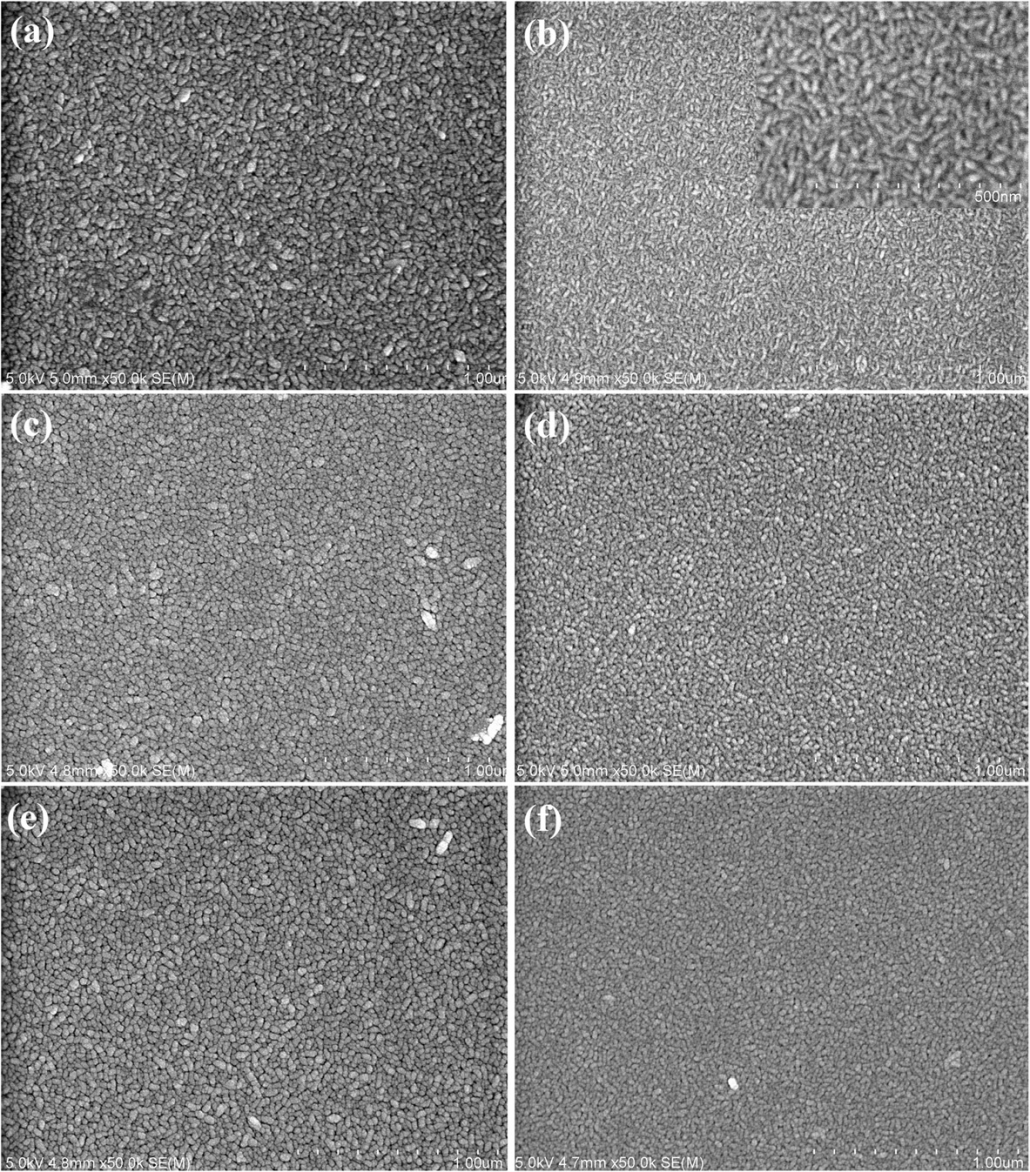
**SEM images. (a)** As-grown ZnO and **(b)** 3 at.% Al-doped ZnO films. High resolution images are also inserted; **(c)** undoped and **(d)** 3 at.% Al-doped films annealed in air; **(e)** undoped and **(f)** 3 at.% Al-doped ZnO film annealed in Ar.

The morphology of the films was observed by SEM. Typical images of undoped and 3 at.% Al-doped films are shown in Figure 3a,b. All films are comprised of uniform elongated grains. Otherwise, some grains of the undoped ZnO film have an inclination angle with the substrate surface. However, the grain sizes are much smaller and the elongated grains are mainly parallel to the substrate surface in Al-doped films. This demonstrates that Al doping would result in ZnO grains growing preferentially with c-axis parallel to the substrate surface. And it is consistent with that indicated in XRD spectra in Figure 1.

**Figure 4:**
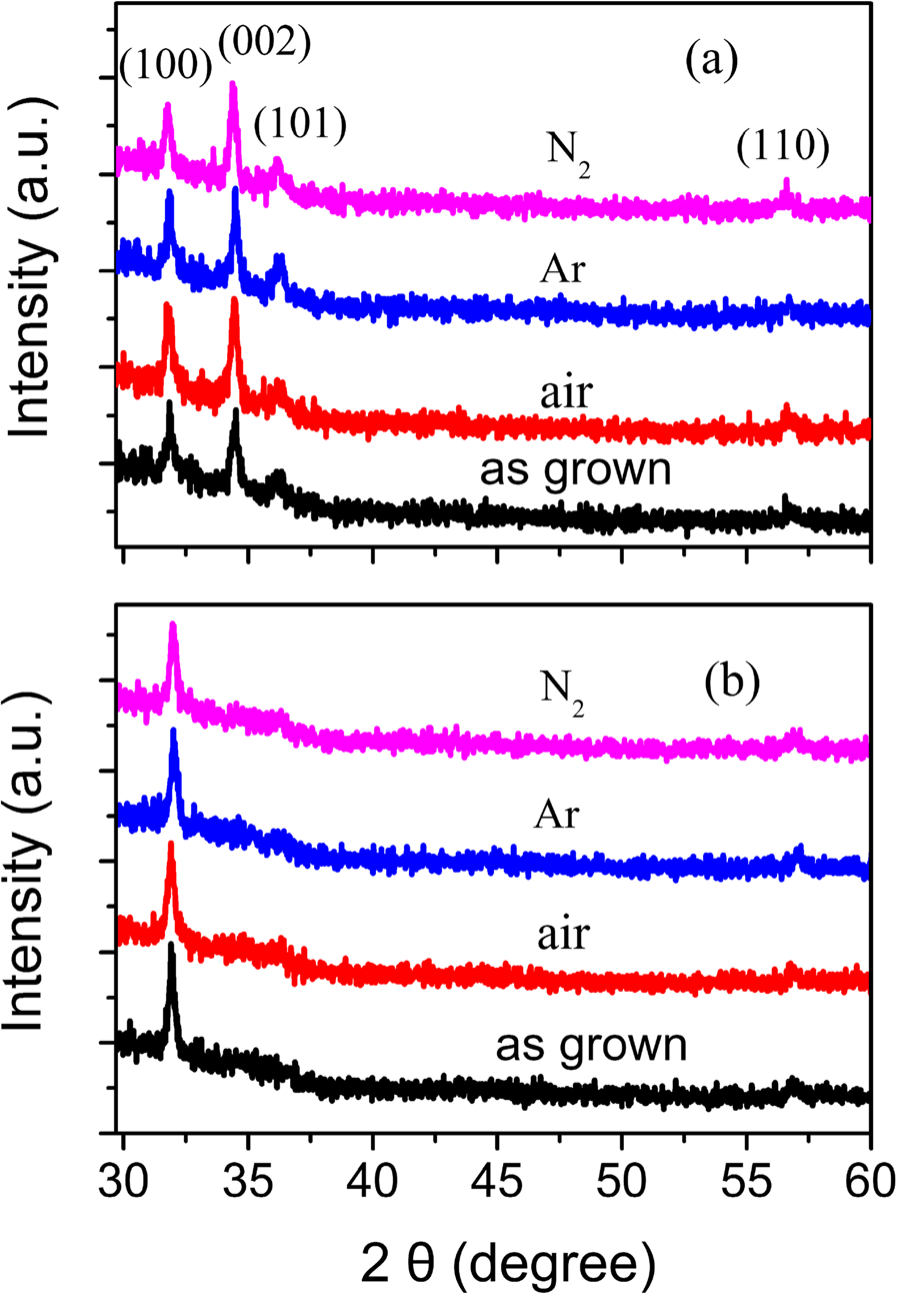
**XRD patterns of films annealed in different ambient. (a)** Undoped and **(b)** 3 at.% Al-doped ZnO.

To investigate the thermal stability of the films, undoped and Al-doped ZnO films were annealed at 350°C in Ar, N_2_, and air ambient for 20 min, separately. The SEM images of the undoped and 3 at.% Al-doped films after annealing are shown in Figure 3c-f. No obvious variation of the grain sizes is observed after annealing, which is similar to that reported by Lin et al. [20]. This indicates that the grains do not grow large by coalescence or coarsening during annealing. The XRD spectra of the undoped and 3 at.% Al-doped films before and after annealing in different atmospheres are shown in Figure 4a,b, respectively. Whatever the annealing atmosphere is, the diffraction intensity of the undoped films increases slightly and the diffraction intensity of (002) planes becomes slightly stronger than that of (100) planes after annealing. This means that the grains in undoped ZnO films recrystallize with c-axis inclining to be perpendicular to the substrate surface. Maeng et al. [15] reported that amorphous phase existed in undoped and Al-doped ZnO films grown by ALD at 60°C to 250°C. Thus, the increase in diffraction intensity of (002) planes in Figure 4a can be attributed to the recrystallization of the amorphous phase in the as-grown films. Different from that observed in undoped ZnO films, the diffraction intensity of (100) planes of Al-doped films decreases slightly after annealing. This indicates that annealing results in the formation of defects or induces local segregation of Al oxide in the doped films, which weakens the diffraction from (100) planes.

### Optical properties of the films

**Figure 5:**
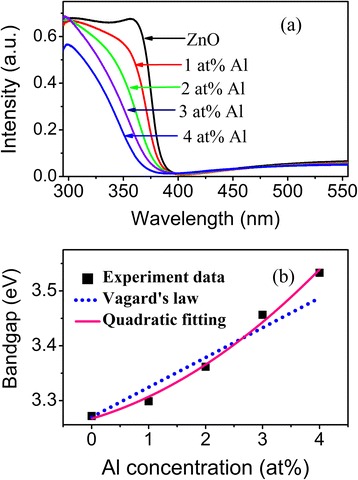
**Absorption spectra and optical bandgap of as-grown films. (a)** Absorption spectra of as-grown undoped and Al-doped ZnO films; **(b)** the variation of ZnO optical bandgap with doped Al concentrations derived by Tauc’s relationship, calculated by Vagard’s law, and fitted by polynomial function.

The absorption spectra of the as-grown undoped and Al-doped films are shown in Figure 5a. Both undoped and Al-doped films have strong absorption in the UV region and good transmittance (>90%) in the visible region. The absorption intensity of the undoped ZnO film increases quickly at the absorption edge, and the absorption peak is obvious; while absorption intensity of Al-doped films increases slowly at the absorption edge, and the absorption peaks become unobvious. This indicates that Al doping degrades the film crystal quality. Otherwise, Al doping leads to a blue shift of the film absorption edge and the shift increases monotonically with an increase in Al concentration. This is similar to that reported in [9-12,20]. Al^3+^ radius is smaller than that of Zn^2+^ [24]. Substitution of lattice Zn^2+^ by Al^3+^ would widen the ZnO bandgap. The blue shift of the absorption edge of the doped films indicates that the doped Al^3+^ ions are located in lattice sites and form Zn_1 −_*x*Al*x*O alloys. For a direct type of a semiconductor, the optical bandgap *Eg* could be estimated from the optical absorption spectra using Tauc’s relationship [5]:

1 $$ \alpha \mathrm{h}\upnu =A\;{\left(E-{E}_g\right)}^{1/2} $$

Where *α* is the absorption coefficient, *E* is the photon energy, and *A* is a constant. The derived optical bandgap of the undoped and Al-doped ZnO films are shown in Figure 5b. The film bandgap widens from 3.27 to 3.53 eV as Al concentration increases from 0 to 4 at.%. The optical bandgap of Al_2_O_3_ is 8.7 eV, and ZnO is 3.27 eV [25] at room temperature. The bandgap of Al-doped ZnO films calculated by Vagard’s law is also shown in Figure 5b. The calculated bandgaps are not consistent with that derived from absorption spectra. Disorders in the alloying could result in a possible aperiodicity in the compound lattice which would produce a bowing effect of the bandgap. Then, the bandgap of a semiconductor alloy (*Eg*) would have a quadratic dependence on the atomic fraction of one compound (*x*) described as [26]:

2 $$ {E}_g=a+bx+c{x}^2 $$

Where *α*, *b*, and *c* are constants. The bandgap of Al-doped ZnO films fitted by the formula (Equation 2) is also shown in Figure 5b. The fitted curve is consistent well with that of that derived from the optical spectra. The dependence of the bandgap (*Eg*) of Al-doped films on Al concentration can be described as:

3 $$ {E}_g=3.27+3.06x+93.50{x}^2 $$

**Figure 6:**
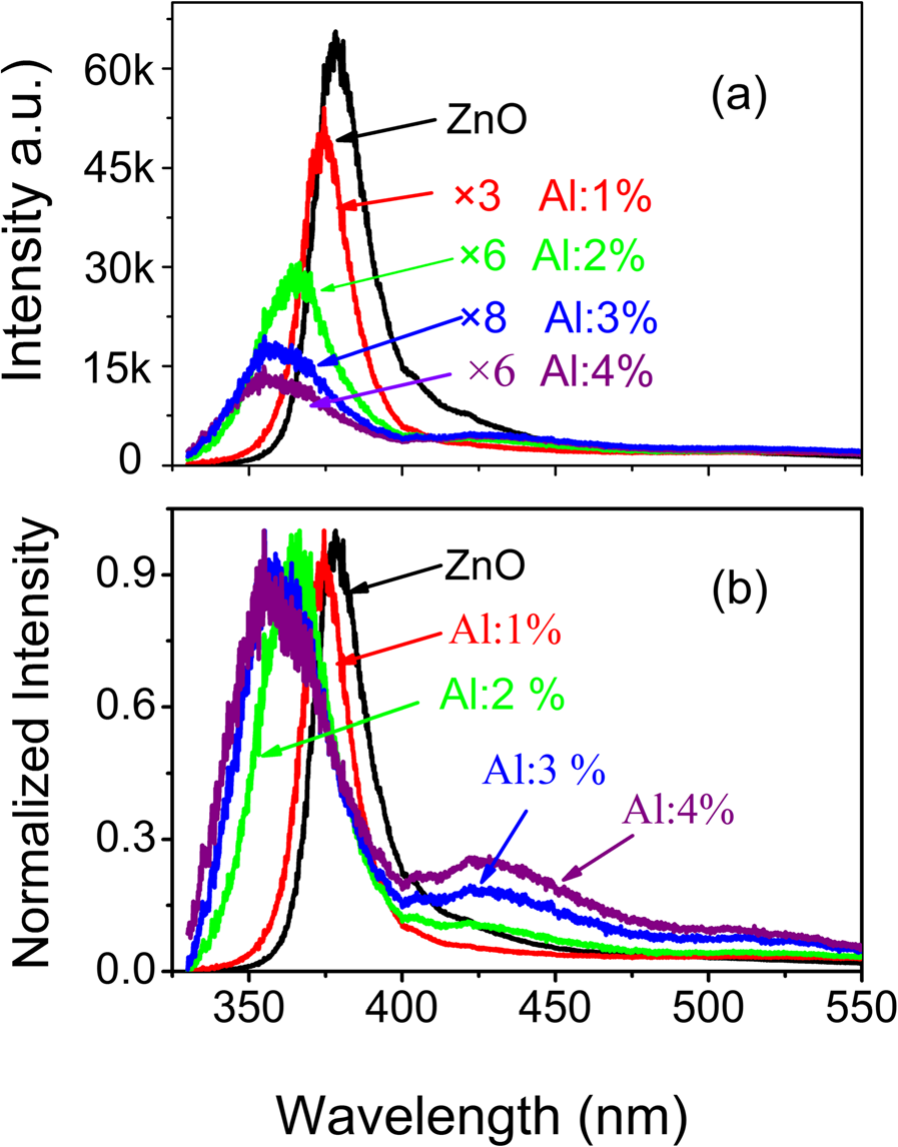
**PL spectra of as-grown films. (a)** PL spectra; **(b)** the normalized PL spectra of as-grown undoped and Al-doped ZnO films.

The emission spectra of the as-grown undoped and Al-doped films are shown in Figure 6a. Emission of undoped ZnO film consists of a strong UV emission peak at 378 nm and a weak blue emission peak as a shoulder at 424 nm. The UV emission is usually ascribed to the emission of bandgap and the blue emission to Zn interstitials [27]. O vacancy emission from 510 to 550 nm is almost unobservable. This indicates that O vacancy concentration in undoped ZnO film grown by ALD is very low. After doping with Al atoms, the UV emission peaks of the films broaden and shift blue obviously. Otherwise, the UV emission intensity decreases dramatically with an increase in Al concentration from 0 to 3 at.%. When Al concentration increases further, the emission keeps almost unchangeable. This suggests that Al doping widens the film bandgap and introduces non-radiative recombination centers or defects as well. To see the evolution of blue shift and defect emission clearly, the normalized PL spectra are shown in Figure 6b. Except the blue shift of the UV emission, the Zn interstitial emission at 424 nm is enhanced and an additional emission at 526 nm is observed in the doped films. Emission at 526 nm is attributed to the emission of O vacancies [28]. The relative intensity of emission at 424 and at 526 nm enhances with an increase in Al concentration. This indicates that Al doping would introduce Zn interstitials and O vacancies. Kim et al. [29] also reported that O vacancies and Zn interstitials could form simultaneously in Al-doped ZnO film.

**Figure 7:**
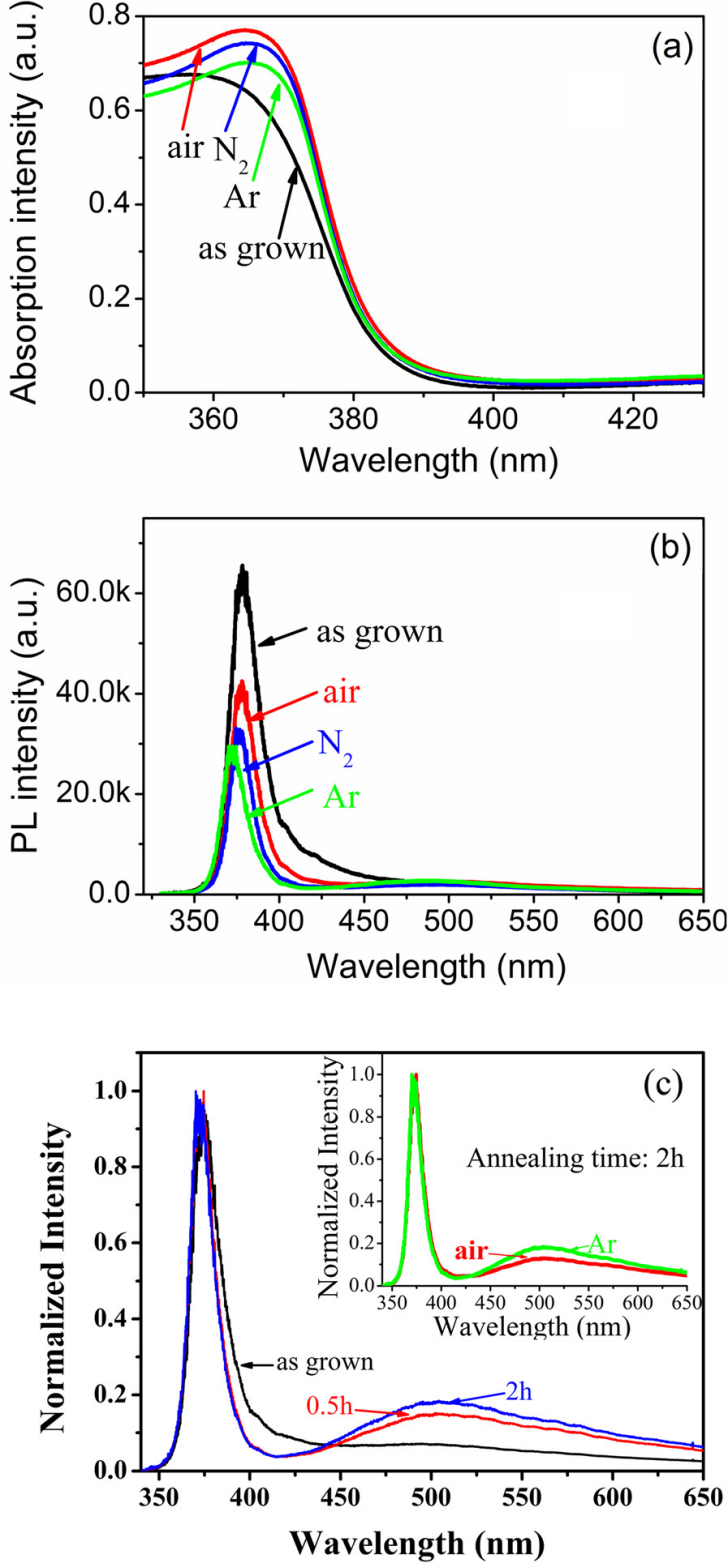
**Optical spectra of undoped ZnO films before and after annealing. (a)** Absorption spectra; **(b)** PL spectra of undoped ZnO films before and after annealing in Ar, N_2_, and air ambient; **(c)** normalized PL spectra of the undoped films after annealing in Ar for different times; normalized PL spectra annealed in Ar or air for 2 h are also inserted.

To investigate the effects of post-annealing on the optical properties of the films, the absorption and PL spectra of the films after annealing in different atmospheres were also measured. The typical absorption spectra of the undoped ZnO film before and after annealing are shown in Figure 7a. Compared with the spectrum of the as-grown film, the absorption intensity around the absorption peak enhances obviously after annealing. Furthermore, the absorption intensity depends on the annealing atmosphere. The absorption intensity of the film is highest after annealing in air, followed by annealing in N_2_, and finally, by annealing in Ar atmosphere. The absorption intensity is proportional to electron state density of valence band and conduction band, which is proportional to total grain volume. The grain sizes are almost unchanged after annealing (Figure 3). Then, the enhancement of the absorption after annealing could be attributed to the effects of structure evolution. During annealing process, the amorphous components would be recrystallized. Improvement of the film crystal quality would lead to an increase in electron state density and then increase the absorption intensity around absorption peak. When the undoped film is annealed in air, O in the atmosphere could be adsorbed on the film surface, which suppresses the production of O vacancy defects and improves the crystal quality. When the undoped film is annealed in Ar atmosphere, O vacancy defects would be introduced and the absorption would be lower as that shown in Figure 7a. The emission spectra of undoped films after annealing are shown in Figure 7b. Compared with that of the as-grown film, the UV emission of the undoped films weakens and has a blue shift after annealing. The weakening and shift are sensitive to the annealing atmosphere. Annealing in air shows the least effect on weakening and blue shift of the UV emission, while annealing in Ar shows the most obvious effect. Otherwise, Zn interstitial emission at 424 nm disappears and the relative intensity of O vacancy emission round at 526 nm increases after annealing. This indicates that Zn interstitials in the as-grown undoped ZnO film could be annealed out, but O vacancies would be introduced simultaneously. The theoretic annealing temperature for Zn interstitials and oxygen vacancies with 2^+^ charge states calculated by Janotti et al. are 216 and 655 K, respectively [30]. The annealing temperature is 350°C (623 K) in this work, and Zn interstitials could be annealed out easily. Zn interstitials are shallow donors. High density of interstitial defects in the as-grown film would narrow the optical bandgap due to overlapping of the defect band and conduction band. Annealing out of Zn interstitials would separate the conduction band and defect band. Then, UV emission energy would be higher after annealing. Otherwise, part of the deficient surface oxygen would be compensated by O in the atmosphere when the film is annealed in air, while O vacancies would be introduced when the film is annealed in Ar. This would result in the difference in UV emission energy and intensity of the films annealed in different atmospheres. To further investigate the effects of annealing on the variation of defects, the normalized PL spectra of the undoped films after annealing in Ar for different time are shown in Figure 7c. As the annealing time increases, the relative intensity of emission at 526 nm enhances. This indicates that the concentration of O vacancies increases with an increase in the annealing time. The normalized PL spectra of films annealed in Ar or air are inserted in Figure 7c. The relative intensity of emission at 526 nm of the film annealed in Ar is stronger than that annealed in air. This phenomenon further indicates that O vacancies would be introduced more easily when the film is annealed in Ar.

**Figure 8:**
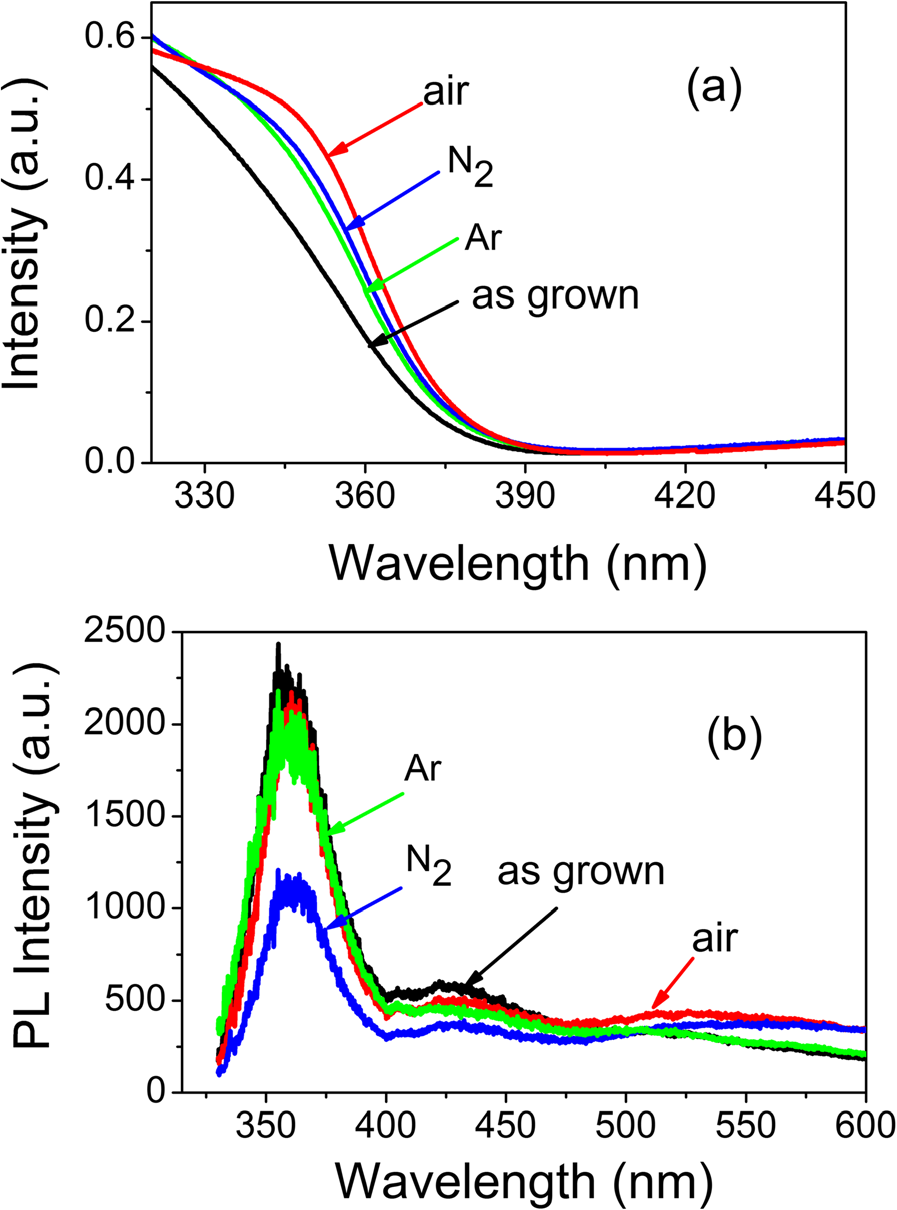
**Optical spectra of 3 at.% Al-doped ZnO films before and after annealing. (a)** Absorption spectra; **(b)** PL spectra of 3 at.% Al-doped ZnO films before and after annealing in Ar, N_2_, and air ambient.

The absorption spectra of 3 at.% Al-doped film before and after annealing in different atmospheres are shown in Figure 8a. Similar to that observed in the undoped film, the absorption intensity around the absorption peak increases obviously after annealing. This also can be ascribed to the effects of the recrystallization of amorphous parts. Otherwise, the absorption peak has a red shift. This indicates that the bandgap of Al-doped films shrinks after annealing. Lattice Al concentrations in the doped films after annealing were calculated with absorption edge values derived from Figure 3 using the formula (Equation 3) and are listed in Table 1. Al concentration in 3 at.% Al-doped film decreases to 2.3 at.% after annealing in Ar and decreases to 2.0 at.% after annealing in air. The shrink of the bandgap is due to the decrease in lattice Al concentration, and lower lattice Al concentration after annealing in air can be attributed to the easy formation of metastable Al oxide phase during annealing. The typical PL spectra of 3 at.% Al-doped films before and after annealing in different atmospheres are shown in Figure 8b. Different from that observed in undoped ZnO films, the UV emission peaks are almost unchanged and UV emission is pinned at 360 nm. This demonstrates that the UV emission of the doped films at high Al concentration is related to the localized shallow traps and the defect states in Al-doped films are much stable.

**Table 1:** **Lattice Al concentration before and after annealing in different ambients**

**Al-doped ZnO**	**Al concentration (at.%)**			
Before annealing	1	2	3	4
Annealed in air	0.3	1.0	2.0	3.0
Annealed in N_2_	0.3	1.2	2.2	3.2
Annealed in Ar	0.3	1.2	2.3	3.4

### Electrical properties of the films

The resistivity, mobility, and carrier concentration of the as-grown undoped and Al-doped ZnO films are shown in Table 2. The carrier concentration increases from 1.4 × 10^19^ to 2.1 × 10^20^ cm^−3^ as Al concentration increases from 0 to 3 at.%. With a further increase in Al concentration, the carrier concentration decreases. The bandgap of 4 at.% Al-doped film is much wider than that of 3 at.% Al-doped film (Figure 5), which means that Al atoms are still in lattice sites in 4 at.% Al-doped film. The decrease of carrier concentration in 4 at.% Al-doped film can be attributed to the production of carrier traps other than the formation of metastable Al oxide phase as that reported in [3,31]. The carrier mobility decreases from 16.4 cm2/Vs in undoped ZnO film to 4.6 cm^2^/Vs in 4 at.% Al-doped film. Similar dependence of carrier mobility on Al concentration has been reported by other groups [10,15,21]. The mobility of carriers has a close relation with the scattering by ionized impurities and grain boundaries [32]. Amorphous components exist in the as-grown films (Figures 4, 7, and 8). It can be supposed that the crystalline grains are surrounded by amorphous components, and there are no definitive grain boundaries in the as-grown films. Thus, the ionized impurity scattering and grain size effect would play dominate roles in the mobility of carriers. Once Al dopes into ZnO, Al atoms occupy Zn lattice sites. They ionize and serve as ionization scatter centers. Otherwise, the grain sizes of Al-doped ZnO films are smaller than that of undoped ZnO film (Figure 3). All these lead to the decrease of carrier mobility in Al-doped ZnO films. It also can be seen from Table 2 that the resistivity of the films decreases from 2.7 × 10^−2^ Ω cm of the undoped ZnO film to 6.1 × 10^−3^ Ω cm of 1 at.% Al-doped film. With a further increase in Al content to 3 at.%, the resistivity decreases slightly to 5.3 × 10^−3^ Ω cm_._ As Al content increases to 4 at.%, the resistivity increases. The minimum resistivity of 5.3 × 10^−3^ Ω cm is still larger than 2.8 × 10^−4^ Ω cm in 2 at.% Al-doped ZnO film grown by PLD [13]. Growth of ZnO films by ALD is induced mainly by self-saturated surface reaction. Otherwise, the films deposited by ALD are usually performed at temperatures much lower than that deposited by PLD. Then, the films deposited by ALD would have lower intrinsic defect density and larger resistivity.

**Table 2:** **Resistivity, carrier concentration, and mobility of the films before and after annealing in different ambients**

**Al (at.%)**	***ρ*** **(Ω • cm)**	***n*** **(cm** ^**−3**^ **)**	***μ*** **(cm** ^**2**^ **/** ***Vs*** **)**	***ρ*** **(Ω • cm)**	***n*** **(cm** ^**−3**^ **)**	***μ*** **(cm** ^**2**^ **/** ***Vs*** **)**
	As-grown			Annealed in air		
0	2.7 × 10^−2^	1.4 × 10^19^	16.40	2.6 × 10^2^	-	-
1	6.1 × 10^−3^	9.7 × 10^19^	10.56	3.2 × 10^1^	-	-
2	6.4 × 10^−3^	1.5 × 10^20^	6.33	2.0 × 10^1^	-	**-**
3	5.3 × 10^−3^	2.1 × 10^20^	5.63	2.1 × 10^1^	-	-
4	7.7 × 10^−3^	1.8 × 10^20^	4.38	4.0 × 10^1^	-	-
	Annealed in Ar			Annealed in N_2_		
0	6.0 × 10^1^	-	-	4.4 × 10^2^	-	-
1	3.1 × 10^−1^	2.2 × 10^19^	0.91	2.1 × 10^0^	5.9 × 10^18^	0.50
2	1.1 × 10^−1^	5.7 × 10^19^	0.98	3.0 × 10^−1^	3.4 × 10^19^	0.60
3	6.8 × 10^−2^	7.8 × 10^19^	1.12	5.0 × 10^−1^	3.2 × 10^19^	0.39
4	1.1 × 10^−1^	5.6 × 10^19^	0.97	1.8 × 10^0^	4.8 × 10^18^	0.70

To reveal the influences of annealing and annealing atmosphere on the film properties, electrical properties of the films after annealing were also measured and the relevant data are listed in Table 2. The conductivity of both undoped and Al-doped ZnO films degrades dramatically after annealing, and the degradation is also very sensitive to the annealing atmosphere. Annealing in air induces the resistivity of both undoped and doped films increase about four orders, and the carrier concentration and mobility cannot be measured within the instrument resolution limitation. Although the resistivity of undoped ZnO film annealed in Ar is lower than that annealed in air or N_2_ ambient, it is still too high (60 Ω cm) to detect the carrier concentration and mobility. However, the conductivity of the Al-doped films is much superior to that of the undoped ZnO film after annealing. The film conductivity annealed in Ar is superior to that annealed in N_2_, which is much better than that annealed in air. Otherwise, the carrier concentration of Al-doped films after annealing in Ar is about one third to a quarter of the as-grown films. However, the mobility of Al-doped films with different Al content is very close. The dependence of carrier concentration on Al concentration shows similar evolution after annealing in N_2_, but the carrier concentration and mobility are lower than that annealed in Ar (Table 2). The low carrier concentration after annealing can be attributed to the decrease of Al concentration in lattice sites (Table 1). Otherwise, nitrogen would adsorb at the grain boundaries during annealing and act as electron traps [33]. All these would result in a decrease in the carrier concentration in the films annealed in N_2_. The close mobility of Al-doped films with different Al contents indicates that scattering of carriers by ionized centers in annealed films is not the dominate factor. The amorphous parts of the films undergo recrystallization during annealing, thus the scattering of carries by grain boundaries becomes important after annealing. According to the Setos model [31], the effective mobility *μ*eff at grain boundaries can be described as:

4 $$ {\mu}_{\mathrm{eff}}=\mathrm{L}\mathrm{e}\;{\left(2\pi {m}_e^{*}kT\right)}^{-1/2}{e}^{-{E}_b/kT} $$

Where *L* is the lateral size of the grain, ${m}_e^{*}$ is the electron effect mass, *T* is the film temperature, and *k* is the Boltzmann constant*. E*_*b*_ is the energy barrier height, which can be expressed as [31]:

5 $$ {E}_b=\frac{e^2{Q}_t^2}{8\varepsilon {\varepsilon}_0n}\cdots \mathrm{f}\mathrm{o}\mathrm{r}\;\mathrm{L}\mathrm{n}>{Q}_t $$

6 $$ {E}_b=\frac{e^2{L}^2n}{8\varepsilon {\varepsilon}_0}\cdots \mathrm{f}\mathrm{o}\mathrm{r}\;\mathrm{L}\mathrm{n}<{Q}_t $$

Where *n* is the carrier concentration, *Q*_*t*_ is the trap density, and *ε*_0_ and *ε* are permittivities of free space and the films. When the concentration of carriers within a grain is greater than the density of the traps at the grain boundary, *E*_*b*_ for carriers to transport would be low and it can be described as Equation 5. Otherwise, *E*_*b*_ would be described as Equation 6. The carrier concentration in undoped ZnO is very low after annealing. Then, the energy barrier height for carriers to transport through the grain boundaries is high. The mobility of carriers is too low to be detected. For Al-doped films annealed in Ar ambient, the carrier concentrations are still around 10^19^ cm^−3^ after annealing. Then, the energy barrier height would be low and the mobility would increase with an increase in carrier concentration (Equations 4 and 5). The measured mobility in Table 2 is consistent well with that predicted by Equation 4. The carrier concentration of Al-doped films annealed in N_2_ is lower than that annealed in Ar ambient. Otherwise, N_2_ absorbed at grain boundaries would act as traps. These lead to an increase in the energy barrier height for carrier to transport through the grain boundaries. The mobility would be lower. The decrease in carrier concentration and mobility give rise to the increase in the resistivity.

## Conclusions

Undoped and Al-doped ZnO films were grown by ALD at 150°C and then annealed at 350°C in Ar, N_2_, and air atmosphere, respectively. The film properties are sensitive to Al concentration and annealing atmosphere. The as-grown films have amorphous components, and annealing induces undoped ZnO to recrystallize preferentially with c-axis perpendicular to the surface. Al doping induces ZnO film growing with c-axis parallel to the substrate surface. It also widens ZnO bandgap, and the bandgap have a quadratic dependence on lattice Al concentration up to 4 at.%. O vacancy concentration is low in the undoped film, and the film has strong UV emission and weak Zn interstitial emission. Annealing would decrease Zn interstitial concentration, introduce O vacancies and non-irradiated centers, and induce a blue shift of the undoped film UV emission, which is sensitive to the annealing ambient. Annealing results in a decrease of lattice site Al concentration, but it has a little effect on the UV emission of Al-doped films. ZnO film resistivity can be decreased to 5.3 × 10^−3^ Ω cm by Al doping. The conductivity of both undoped and Al-doped ZnO films degrades dramatically after annealing, and the degradation is very sensitive to the annealing ambient. The conductivity of Al-doped films annealed in Ar is superior to that in annealed N_2_ atmosphere, which is much better than that annealed in air.

## Abbreviations

ALD: atomic layer deposition
TCO: transparent conducting oxide
ITO: indium tin oxide
DEZ: diethylzinc
TMA: trimethylaluminum
MS: magnesocene
XRD: X-ray diffractometer
SEM: scanning electron microscope
PL: photoluminescence
